# Function of P2X4 Receptors Is Directly Modulated by a 1:1 Stoichiometric Interaction With 5-HT_3_A Receptors

**DOI:** 10.3389/fncel.2020.00106

**Published:** 2020-05-05

**Authors:** Paola Soto, Pablo S. Gaete, Christian Fuentes, Benjamin Lozano, Pamela A. Naulin, Xavier F. Figueroa, Nelson Patricio Barrera

**Affiliations:** Department of Physiology, Faculty of Biological Sciences, Pontificia Universidad Católica de Chile, Santiago, Chile

**Keywords:** receptor-receptor interaction, atomic force microscopy, stoichiometry, P2X4 receptor, 5-HT_3_A receptor, intracellular Ca^2+^, ATP

## Abstract

Interacting receptors at the neuronal plasma membrane represent an additional regulatory mode for intracellular transduction pathways. P2X4 receptor triggers fast neurotransmission responses *via* a transient increase in intracellular Ca^2+^ levels. It has been proposed that the P2X4 receptor interacts with the 5-HT_3_A receptor in hippocampal neurons, but their binding stoichiometry and the role of P2X4 receptor activation by ATP on this crosstalking system remains unknown. *Via* pull-down assays, total internal reflection fluorescence (TIRF) microscopy measurements of the receptors colocalization and expression at the plasma membrane, and atomic force microscopy (AFM) imaging, we have demonstrated that P2X4/5-HT_3_A receptor complexes can interact with each other in a 1:1 stoichiometric manner that is preserved after ATP binding. Also, macromolecular docking followed by 100 ns molecular dynamics (MD) simulations suggested that the interaction energy of the P2X4 receptor with 5-HT_3_A receptor is similar at the *holo* and the *apo* state of the P2X4 receptor, and the interacting 5-HT_3_A receptor decreased the ATP binding energy of P2X4 receptor. Finally, the P2X4 receptor-dependent Ca^2+^ mobilization is inhibited by the 5-HT_3_A interacting receptor. Altogether, these findings provide novel molecular insights into the allosteric regulation of P2X4/5-HT_3_A receptor complex in lipid bilayers of living cells *via* stoichiometric association, rather than accumulation or unspecific clustering of complexes.

## Introduction

Receptor-receptor interaction has been an extensive research area with enormous implications in neuronal crosstalking transduction mechanisms, which usually provide new molecular ways to tune and regulate the receptor function (Barrera et al., 2005b; Ferre et al., 2007). Ionotropic receptors located at the central nervous system, such as those belonging to the cys loop family, have been found to form macromolecular complexes composed by two or more different receptors (Ferre et al., 2007). In particular, P2X2 and 5-HT_3_A receptors are co-expressed at the plasma membrane and physically interact with each other in myenteric neurons and heterologous systems. This interaction depends on the second intracellular loop of the 5-HT3A subunit and an unknown region of the P2X2 receptor that was initially thought to be the C-term tail (Boué-Grabot et al., 2003). As a functional consequence, the inhibition of the additive responses attained with serotonin and ATP was observed. Similar receptor-receptor interactions and inhibitory crosstalking have been observed between P2X and nicotinic acetylcholine (nACh) receptors (Barajas-López et al., 1998) and between P2X and GABA receptors (Jo et al., 2011). Recently, Emerit et al. (2016) showed that the 5-HT_3_A receptor can colocalize with the P2X4 receptor at the plasma membrane of hippocampal neurons. Nevertheless, it remains unknown the stoichiometry of these interacting receptors, whether the interaction is controlled by the agonist binding and the functional role of this regulatory mechanism.

P2X4 receptor is also regulated by direct interactions with phospholipids. In this context, phosphoinositides PI(4,5)P2 (PIP_2_) and PI(3,4,5)P3 (PIP_3_) can modulate P2X4 activity, apparently, by direct interactions with the proximal C-terminal domain of the receptor (C360–V375), which was shown to be required for the development of full receptor function, since depletion of PIP_2_ and PIP_3_ dramatically inhibits the P2X4-mediated Ca^2+^ signal activated by ATP (Bernier et al., 2008). Therefore, as it has been proposed that 5-HT_3_A receptor may interact with the C-term tail of P2X receptors (e.g., P2X2 receptor), the potential interaction between this receptor and P2X4 receptor may also represent a relevant control mechanism of the phospholipids-mediated P2X4 regulation.

A variety of methods have been continuously developed to tackle the molecular architecture of interacting membrane proteins, from high resolution, such as x-ray crystallography, to complementary biophysical approaches, including cryo-electron microscopy, mass spectrometry and atomic force microscopy (AFM). Based on the surface scanning of the sample, AFM has already been used to characterize the molecular architecture of individual P2X and 5-HT3 receptors (Barrera et al., 2005a,c, 2007; Antonio et al., 2011),among others.

Herein, *via* pull-down assay of interacting receptors, AFM imaging, macromolecular docking, molecular dynamics (MD) simulations, and total internal reflection fluorescence (TIRF) microscopy analysis, we propose that P2X4 receptor physically interacts in a 1:1 stoichiometric manner with 5-HT_3_A receptor, which is maintained after ATP binding. By measurements of intracellular Ca^2+^ levels, we further confirmed that the interacting 5-HT_3_A receptor inhibits the response to ATP of the P2X4 receptor. Altogether, these findings provide insights into the inhibitory responses triggered *via* stoichiometric binding of interacting receptors, which consequently support the notion that interacting receptors in specific numbers rather than receptor aggregation are involved in crosstalking neuronal responses.

## Materials and Methods

### Expression of P2X4/5-HT_3_A Receptor Complexes on tsA201 Cells

tsA201 cells (Sigma–Aldrich, St. Louis, MO, USA. Cat. # 96121229-1VL) were grown in DMEM medium (Gibco, Grand Island, NY, USA), supplemented with 10% fetal bovine serum (FBS, Gibco, Grand Island, NY, USA), 100 unit/ml penicillin (Sigma–Aldrich, St. Louis, MO, USA) and 100 μg/ml streptomycin (Sigma–Aldrich, St. Louis, MO, USA). Cells were maintained at 37°C in a humidified 5% CO_2_–95% air atmosphere incubator. To induce the expression of P2X4 and 5-HT_3_A receptors, tsA201 cells with a confluence of 40–60% were transfected with the pDNAs of P2X4 and 5-HT_3_A receptors using polyethyleneimine (PEI, Sigma–Aldrich, St. Louis, MO, USA) as transfection reagent. Briefly, 10 ml of DMEM without serum were mixed with 275 μl of 1 mg/ml PEI alone (mock transfection) or plus pDNAs for P2X4 and/or 5-HT_3_A (25 μg each) and left for 15 min at room temperature. Then, cells were incubated with this mixture for 24 h at 37°C. For transfection, the following constructs were used; rat P2X4 (*Rattus norvegicus*, GenBank: U47031.1) with a C-terminal hemagglutinin (HA) tag and human 5-HT_3_A (*Homo sapiens*, GenBank: AK304630.1) with a C-terminal MYC/His-6 epitope tag, subcloned into the vector pcDNA3.1 (Invitrogen). Immunofluorescence analysis and Ca^2+^ measurements were performed using cells seeded onto sterile glass coverslips.

### Measurements of Intracellular Ca^2+^ Levels

Ca^2+^ measurements were performed using the fluorescent Ca^2+^ indicator, Fluo 4, as described recently by Lillo et al. (2018). To upload Fluo 4, cells were incubated with 5 μM Fluo 4-AM for 45 min in a MOPS-buffered solution (composition in mM: 118 NaCl; 5.4 KCl; 2.5 CaCl_2_; 1.2 KH_2_PO_4_; 1.2 MgSO_4_; 11.1 glucose and 5 MOPS) adjusted to pH 7.4. The fluorescent indicator was washed out and the experiments started after 15 min of equilibration. Cells were visualized using an Olympus BX50 WI microscope and the fluorescent signal was recorded using an intensified CCD camera (Retiga Fast 1394, QImaging) and IPLab software. Images were acquired every 2 s at basal conditions and during 2 min of stimulation. Changes in intracellular Ca^2+^ levels were expressed as the variations of the fluorescence intensity along the time (F/F_0_), where F is the fluorescence detected during the recording and F_0_ is the basal fluorescence, or the maximum net fluorescence observed after stimulation (ΔF/F_0_). Three concentrations of ATP (1, 10 and 100 μM) were tested and, to confirm the activation of P2X4 receptors, 30 μM PPADS was preincubated during 15 min before the ATP application. Both reagents were poured over the cultured cells.

### Confocal and Epifluorescence Microscopy

Cells were fixed with 1% formaldehyde and then washed with PBS solution (composition in mM: 136.9 NaCl; 2.68 KCl; 10.44 NaH_2_PO_4_; 1.76 KH_2_PO_4_) adjusted to pH 7.4. Nonspecific protein binding sites were blocked with PBS containing 0.1% FBS. Cells were incubated overnight with an anti-MYC monoclonal antibody to detect 5-HT_3_A receptor (1:250, Thermo Fisher Scientific, Cat. # PA1-981, Rockford, IL, USA) and an anti-HA monoclonal antibody to label P2X4 receptor (1:250 Thermo Fisher Scientific, Cat. # 26183, Rockford, IL, USA). Cells were washed three times for 10 min with PBS and then incubated for 1 h with a secondary antibody conjugated to Alexa Fluor^®^555 for anti-MYC (1:500, Cat. #A-21424, Molecular Probes, Eugene, OR, USA) or Alexa Fluor^®^488 for anti-HA (1:500, Cat. # A-11029, Molecular Probes, Eugene, OR, USA). Then, cells were washed and mounted with Fluoromount-G (Electron Microscopy Sciences, Cat. # 17984-25, Hatfield, PA, USA). The fluorescent signal was examined using either an Olympus IX81 confocal inverted microscope coupled with an ORCA R2 Hamamatsu CCD camera or an Olympus BX41 WI microscope coupled with a Jenoptik ProgRes C5 CCD camera. As a negative control, primary antibodies were omitted.

### TIRF Microscopy

TIRF microscopy imaging was performed using a NIKON Eclipse Ti2-E microscope with the module NIKON H-TIRF with a 100X magnification and up to 100 nm sample depth. Sample preparation was similar to the confocal experiments except for using PBS as mounting protocol and secondary antibodies conjugated to Alexa Fluor^®^555 and Alexa Fluor^®^488 to identify anti-HA and anti-MYC primary antibodies, respectively.

### Quantification of P2X4 Receptor Expression and Colocalization of P2X4/5-HT_3_A Receptor Complexes

Fluorescence intensity of P2X4 receptor expression attained by TIRF and epi microscopies were quantified using the ImageJ software (Schneider et al., 2012). Window and level parameters were optimized to select the Regions of Interest (ROIs) and exclude background noise. Fluorescence intensity was measured as the mean intensity of each ROI in raw images ± SE. For the colocalization analysis of P2X4/5-HT_3_A receptor complexes, coloc2 plug-in implemented in ImageJ allowed to evaluate the correlation between the pixel’s intensity on each channel *via* Pearson’s R coefficient. Note that protein colocalization and expression experiments were performed using the same instrumental acquisition parameters such as light intensity and exposure time for all the images.

### Purification of P2X4/5-HT_3_A Receptor Complexes

Cells (five flasks of 150 cm^2^ for each condition) were washed with HBS solution (composition in mM: 50 HEPES; 100 NaCl; 2 EDTA) adjusted to pH 7.6, and then, removed by shaking. Cells were collected in falcon tubes and centrifuged at 6,500 g at 4°C for 5 min. The pellet was resuspended in a solubilization solution (9 ml, composition in mM: 10 Tris-HCl; 100 NaCl; 5 EDTA, adjusted to pH 7.6), 1% Triton X-100 (Sigma–Aldrich, Cat. # 9002-93-1), a protease inhibitor mixture (complete, EDTA-Free, Roche) and 10 μl PMSF. The sample was incubated on a rotating wheel for 1 h at 4°C. The supernatant was placed in a Beckman centrifuge tube and subjected to ultracentrifugation at 50,000 *g* at 4°C for 1 h. The supernatant, corresponding to the positive control for the plasma membrane protein and named MEMBRANE fraction in Western blot analysis, was mixed with pre-washed anti-HA agarose beads (Thermo Fisher Scientific) and incubated for 3 h at 4°C. Beads were washed with 10 ml washing buffer (solubilization buffer containing 1% w/v Triton X-100) and centrifuged at 6,500 *g* three times. One last washing step was performed including a solution of 0.1% CHAPS (3-[(3-Cholamidopropyl)dimethylammonio]-1-propanesulfonate; Cat. # 220201, Calbiochem). Finally, proteins were eluted from beads by incubating them with 200 μl of 0.1% CHAPS plus 6 μl HA peptide (Thermo Fisher Scientific). An identical purification protocol was followed when tsA201 cells were expressing only P2X4 receptors. The ELUTION fraction (200 μl), representing the purified receptor or complex, was stored to perform Western blot analysis and AFM imaging. Five independent purification series were performed for the complex. Note that 100 μM ATP was applied in the eluted sample in solution during 30 min at room temperature, before protein adsorption (50 μl) onto mica.

When P2X4/5-HT_3_A receptor complexes were pulled down *via* His6 tag, the purification procedure was similar to that used with the HA tag, except that in this case HisPur Ni-NTA agarose beads were used (Thermo Fisher Scientific) to carry out the extraction of His6 tagged proteins. Later, beads bound to the 5-HT_3_A-MYC/His6 receptors were deposited in purification columns (Cat. # 450015, Invitrogen), and 200 mM imidazole was used for elution. An identical protocol was followed in tsA201 cells expressing only 5-HT_3_A receptors. Five independent purification series were performed for each receptor.

### Western Blot Analysis

Purified proteins were separated by 10% SDS-PAGE and transferred onto a nitrocellulose membrane (BioRad, CA, USA). Primary antibodies: anti-MYC (1:500, Thermo Fisher Scientific, Cat. # PA1-981, Rockford, IL, USA), or anti-HA (1:500 Thermo Fisher Scientific, Cat. # 26183, Rockford, IL, USA) were incubated overnight in a PBS solution containing 4% milk at 4°C. HRP-labeled secondary antibodies (1:1,000, Cat. # 621040, Molecular Probes, Eugene, OR, USA) were incubated at room temperature for 1 h in a PBS solution containing 4% milk. The SuperSignal^®^ West Femto chemiluminescent substrate (Thermo Fisher Scientific) was used to detect protein bands. Molecular mass was estimated with prestained markers (Bio-Rad Laboratories, Hercules, CA, USA). The MyECL™ Imager (Thermo Fisher Scientific) was used to reveal the membrane.

### AFM Imaging

Purified protein samples (50 μl from eluted samples) were placed on muscovite micas (Electron Microscopy Sciences, Cat. # 71855-01, Hatfield, PA, USA) to be adsorbed. After incubation for 15 min at room temperature, the mica was washed with Milli-Q water and dried with nitrogen gas. Images of dry samples were acquired in an AFM (MFP-3D-SA Asylum Research, CA, USA) using the intermittent contact mode. Cantilever was used with a drive frequency of ~300 kHz and a spring constant of 40 N/m. The force applied to obtain the images was kept as low as possible. The target amplitude was ~0.5 V and amplitude setpoint ~0.4 V. Each image (4 μm^2^) was obtained in 15 min and 50 images were analyzed. The molecular volumes of the adsorbed receptor and complex particles were determined from the height and radius of the particle obtained by AFM (Barrera et al., 2008). The molecular volume was calculated from the equation:

(1) $$\text{Vm = (}\pi \text{h/6) }{\text{(3r}}^{2}+{\text{h}}^{2})$$

where h is the height of the particle and r is the radius at half the height. The equation considers the protein molecule as a sphere. Histograms of molecular volumes were fit by Gaussian distribution.

To calculate the individual receptor or complex concentration for the analyzed samples, the following equation was used:

(2) $$\text{M = }\frac{\text{total mica area (}\mu {\text{m}}^{2})\times \text{particle number per scanning area }(\frac{particle}{\mu {\text{m}}^{2}})\times 10^{6}(\frac{\mu l}{L})}{\text{elution volume in mica }(\mu l)\times \text{ Avogadro Number }(\frac{particle}{mol})}$$

where M represents the molarity of the purified particle sample obtained from AFM imaging. For P2X4 receptors, 5-HT_3_A receptors and P2X4/5-HT_3_A receptor complexes, molecular volume cut-offs corresponding to their peaks were 403 and 431 (Antonio et al., 2011), 726 and 788 (Barrera et al., 2005a), and 991 and 1209 (Figure 3), respectively. Calculated values are derived from five independent purification processes for each sample, where all the samples were adsorbed in the same mica type surface during 15 min.

**Figure 1:**
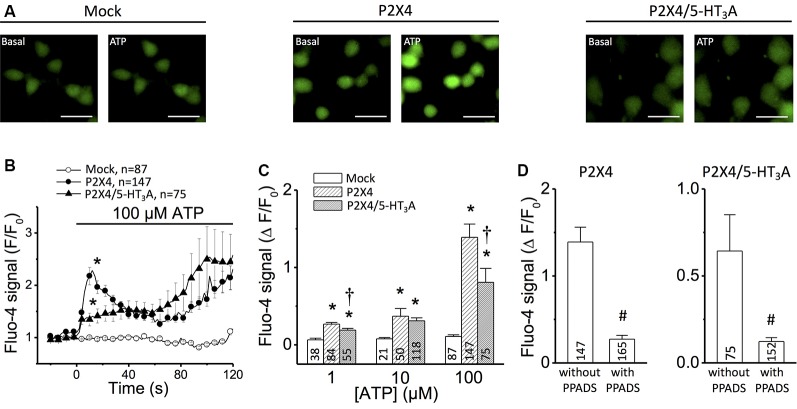
Ca^2+^ measurements in tsA201 cells expressing P2X4 receptor alone or P2X4/5-HT_3_A receptor complexes.** (A)** Representative images of Ca^2+^ measurements in mock-transfected cells, cells transfected with P2X4 receptor alone and cells co-transfected with P2X4 plus 5-HT_3_A receptors. Changes in intracellular Ca^2+^ concentration were detected using the Ca^2+^ indicator Fluo-4. Images were captured before (Basal) and 20 s after the addition of 100 μM ATP. Scale bars represent 30 μm.** (B)** Time course of Fluo-4 fluorescence signal in cells stimulated with 100 μM ATP. The horizontal bar indicates the stimulation period. **(C)** Analysis of the maximum increase in intracellular Ca^2+^ levels observed after stimulation with 1–100 μM ATP. **(D)** Analysis of the maximum increase in intracellular Ca^2+^ levels observed in cells transfected with P2X4 receptor alone (left) or co-transfected with P2X4 and 5-HT_3_A receptors (right) after application of 100 μM ATP in the absence or presence of 30 μM PPADS, an inhibitor of P2X receptors. Three independent Ca^2+^ measurements were carried out for each condition from different batches of cells. Numbers inside bars indicate the number of cells analyzed. Values are the mean ± SE. *p < 0.05 vs. Mock by 1-way ANOVA plus Newman–Keuls *post hoc* test; ^†^*p* < 0.05 vs. P2X4 by 1-way ANOVA plus Newman–Keuls *post hoc* test; ^#^*p* < 0.05 vs. without PPADS by unpaired student’s *t*-test.

**Figure 2:**
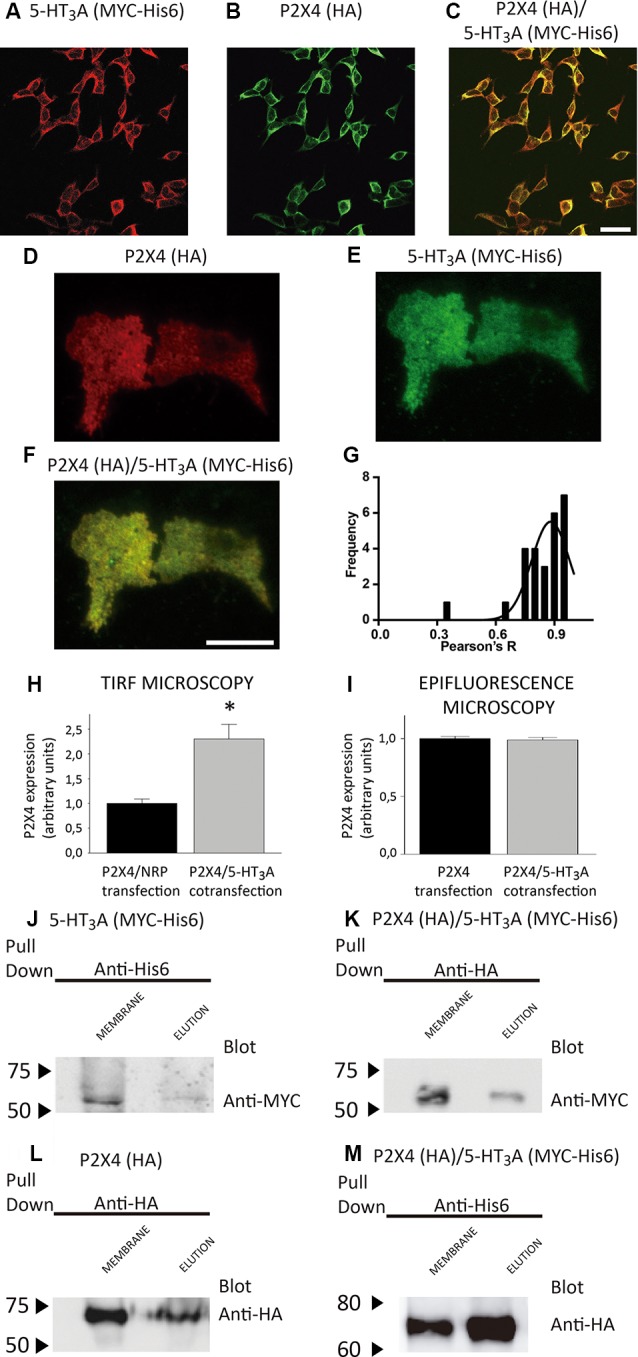
Expression and purification of P2X4/5-HT_3_A receptor complexes. **(A,B)** Detection of co-expressed 5-HT_3_A **(A)** and P2X4 **(B)** receptors by confocal immunofluorescence in tsA201 cells *via* anti-MYC (1:250) and anti-HA (1:250) antibodies, followed by their corresponding secondary antibodies conjugated to Alexa Fluor^®^555 and Alexa Fluor^®^488, respectively. The merge of panels **(A,B)** is shown in **(C)**, where scale bar represents 30 μm. **(D,E)** Analysis by total internal reflection fluorescence (TIRF) microscopy of co-expressed P2X4 **(D)** and 5-HT_3_A **(E)** receptors individually and merge images **(F)** in tsA201 cells *via* anti-HA (1:250) and anti-MYC (1:250) antibodies, followed by their corresponding secondary antibodies conjugated to Alexa Fluor^®^555 and Alexa Fluor^®^488, respectively. Scale bar in **(F)** represents 3 μm.** (G)** Colocalization analysis of the P2X4/5-HT_3_A receptor complexes in tsA201 cells (*n* = 26 from two independent experiments). **(H,I)** P2X4 receptor expression analysis in tsA201 cells through TIRF (**H**, *n* = 26 for P2X4/NRP and P2X4/5-HT_3_A transfections from two independent experiments) and epifluorescence (**I**, *n* = 273 for P2X4 and 254 for P2X4/5-HT_3_A transfections from two independent experiments) measurements. Non-related plasmid (NRP) corresponds to pDNA IRES-GFP (0.5 μg each well) used in the same amount as the other receptor plasmids. Data are shown as mean ± SE. *p < 0.05 vs. P2X4/NRP expression by unpaired *t*-test. **(J,L)** Western blot analysis of the purified samples from tsA201 cells expressing only 5-HT_3_A **(J)** or P2X4 **(L)** receptors identified by anti-MYC or anti-HA antibodies, respectively. Specific bands in plasma membrane enriched fraction (MEMBRANE) and purified (ELUTION) samples were observed at 55 kDa **(J)** for 5-HT3A and 70 kDa **(L)** for P2X4 subunits. **(K,M)** Western blot analysis of purified samples from tsA201 cells co-expressing 5-HT_3_A and P2X4 receptors after pulling down the complexes with either the HA-tag purification **(K)** or His6-tag purification **(M)** method. Bands corresponding to 5-HT3A (anti-MYC antibody; **K**) and P2X4 subunits (anti-HA antibody; **M**) were detected at similar molecular weights compared to those present after single transfection. Arrowheads indicate molecular mass markers in kDa.

**Figure 3:**
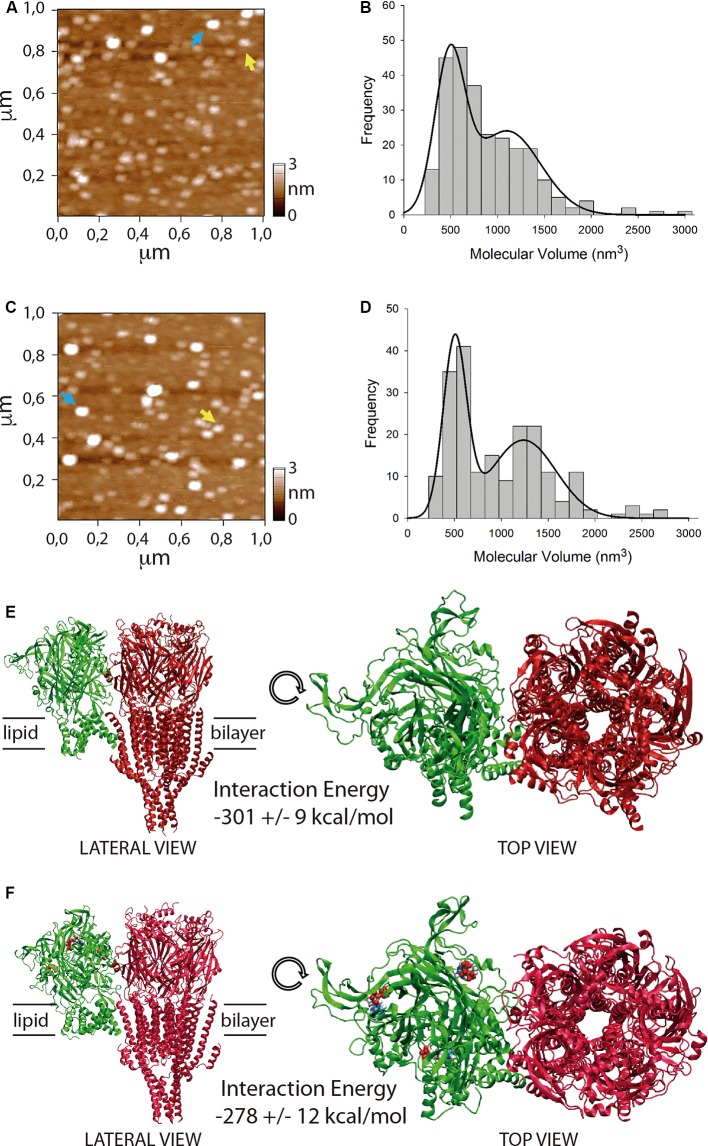
Stoichiometry and interaction energy on P2X4/5-HT_3_A receptor complexes. **(A–D)** Atomic force microscopy (AFM) imaging of purified P2X4/5-HT_3_A receptor complexes in the absence **(A,B)** and presence **(C,D)** of 100 μM ATP. Yellow and blue arrows indicate molecular volumes of 493 nm^3^ and 1,194 nm^3^ **(A)** and 486 nm^3^ and 1,125 nm^3^ **(C)**. Histogram of molecular volumes of particles fitted by double Gaussian peaks in the absence **(B)** and presence **(D)** of 100 μM ATP. **(E)** Molecular docking of the complex formed between P2X4 receptor *apo* (green) and 5-HT_3_A receptor (red). **(F)** Molecular docking of the complex formed between P2X4 receptor *holo* (green) and 5-HT_3_A receptor (red). ATP molecules are depicted as colored blobs. Interaction energy values for both complexes are indicated as mean ± SE after 100 ns molecular dynamics (MD) simulations.

### Molecular Docking and Molecular Dynamics Simulations of the P2X4/5-HT_3_A Receptor Complex

The amino acid sequence of rat P2X4 subunit was obtained from UNIPROT (entry P51577) and aligned against the sequence of the zebrafish crystallized structures of P2X4 receptor (Hattori and Gouaux, 2012; PDB codes 4DW0 for the *apo* structure and 4DW1 for the *holo*-structure) by using MultAlin (Corpet, 1988). One hundred homology models for the *apo* and *holo*-structures were generated by using Modeller 9.18 (Webb and Sali, 2017) and the one having lowest DOPE score for each case was selected and verified by using the web servers MolProbity (Chen et al., 2010) and ProSA (Wiederstein and Sippl, 2007) to evaluate both stereochemical and energetics of each model. From the two final models, N and C-term tails were cut from MET 1 to VAL 28 and from LEU 358 to GLU 388, respectively.

The amino acid sequence of the human 5-HT3A subunit was obtained from UNIPROT (entry P46098) and aligned against the sequence of mouse 5-HT_3_A receptor structure (Basak et al., 2018; PDB code 6BE1). One hundred homology models for the *apo* structures were generated by using Modeller 9.18 (Webb and Sali, 2017) and the one having the lowest DOPE were selected and verified by using the web servers MolProbity and ProSA to evaluate both stereochemical and energetics. From the final model, the N-term tail was cut from MET 1 to THR 30 and the intracellular loop consisting from CYS 356 to ALA 411 was cut as well.

Docking of ATP into the P2X4 receptor was performed in AutoDock (Morris et al., 2008). Binding pockets had volumes of 22.5 × 22.5 × 22.5 Å^3^ and the center of each box was placed as the ligand is seen in the crystal structure. The 100 best conformations were saved and grouped in clusters of root mean square displacement (RMSD) less than 2 Å. Selected dockings for each binding pocket had a negative binding energy and a similar spatial orientation as the crystallized bound ATP.

Due to pentameric and trimeric symmetry of both 5-HT_3_A and P2X4 receptors, respectively (Hattori and Gouaux, 2012; Basak et al., 2018), there are several possible combinations depending upon their interaction surface. However, we used two experimental evidence to take an appropriate orientation for the receptor complex: (1) choosing the minimal distance between both receptors at the second intracellular loop of the 5-HT_3_A receptor, which has been postulated as a domain involved in the physical P2X2/5-HT_3_A receptor interaction (Emerit et al., 2016); and (2) rotating, in the perpendicular plane of the bilayer, all possible conformations of the complex until the largest interaction surface was found since it has been shown that bigger interaction surfaces are more energetically favorable between macromolecular complexes (Casuso et al., 2012). To have a similar comparison between the two dockings, the P2X4 receptor *apo* structure was aligned to the first resulting docking of the P2X4 *holo*/5-HT_3_A receptor complex.

The homology model of the P2X4 receptor in its *holo* state was combined with three ATP molecules in their binding pocket. Later both *apo* and *holo*-structures in the designed position were merged with the structure of the 5-HT_3_A receptor and were protonated accordingly to the physiological pH. Both complexes were inserted in a pre-equilibrated POPC bilayer (of size 204 × 124 Å^2^) created using the Visual Molecular Dynamics suite (VMD; Humphrey et al., 1996). To do this, both the bilayer and the receptor complex were aligned and all overlapped lipids (nearer than 0.8 Å) and water molecules (nearer than 3 Å) were deleted. The complex of receptor-bilayer was solvated by using the TIP3 water model and NaCl was added to both neutralize and provide a physiological concentration of salt. Simulations were performed using NAMD 2.12 with the temperature at 310 K and pressure at 1 atm with periodic boundary conditions (PBC). The first step was to minimize the energy by simulating 10,000 steps in the NVT ensemble, and then, the NPT ensemble was simulated. One hundred ns MD simulations for both P2X4 *apo*/5-HT_3_A and P2X4 *holo*/5-HT_3_A receptor complexes were carried out, which correspond to a similar simulation time already applied in our group to study allosteric regulation of P2X4 receptor by ivermectin (Latapiat et al., 2017). To maintain temperature and pressure constant, Langevin dynamics and Nosé–Hoover Langevin piston methods were used for temperature and pressure coupling. To calculate electrostatic interactions, Ewald sums were used with a grid density of 1 Å. Ligand parameterization (ATP) was done using the SwissParam webserver (Zoete et al., 2011) and the CHARMM27 forcefield was used for lipids and protein. Electrostatic and van der Waals interactions between both receptors (or between P2X4 receptor and ATP molecules) within 12 Å cut off, corresponding to the total interaction energy, were measured by using VMD plugin “NAMDEnergy” every 40 ps during the simulation.

### Reagents

MOPS, ATP and all chemicals of analytical grade were obtained from Sigma–Aldrich (St. Louis, MO, USA). Fluo 4-AM was purchased in Life Technologies (Eugene, OR, USA) and pyridoxalphosphate-6-azophenol-2′,4′ disulfonic acid (PPADS) in Tocris Bioscience (Ellisville, MO, USA). ATP and PPADS were dissolved in water. Fluo 4-AM was dissolved in DMSO.

### Statistical Analysis

Values are represented as means ± SE. Comparisons between groups were made using unpaired student’s *t*-test or 1-way ANOVA plus Newman–Keuls *post hoc* test, as appropriate. A value of *p* < 0.05 was considered significant.

## Results

First, the P2X4 receptor function in transfected tsA201 cells was evaluated by measuring the changes in free intracellular Ca^2+^ concentration observed in response to ATP application. Stimulation with 100 μM ATP evoked a Ca^2+^ signal that showed two components: a first fast-transient component that peaked at ~12 s and decreased thereafter, and a second slow component that starts after ~60 s (Figures 1A,B). In tsA201 cells co-transfected with P2X4 and 5-HT_3_A receptors, the transient increase in intracellular Ca^2+^ was blunted (Figure 1B) whereas the time course of the second component was similar to that observed in P2X4 transfected cells. Although the Ca^2+^ response was evaluated at a range of ATP concentrations (1–100 μM), the most striking difference in the initial Ca^2+^ signaling (first 60 s) between cells transfected with P2X4 receptor alone or co-transfected with P2X4 and 5-HT_3_A receptors was attained with 100 μM ATP (Figure 1C). As expected, the intracellular Ca^2+^ concentration was not affected by ATP in non-transfected cells (mock, Figures 1B,C). Consistent with the activation of P2X4 receptors, the response induced by ATP was almost completely blocked (~85%) by 15 min pre-incubation with the preferential P2X receptor antagonist PPADS (Figure 1D; Lê et al., 1998). Interestingly, the response of co-transfected cells was inhibited by PPADS in the same proportion to that observed in cells transfected with P2X4 alone. As PPADS blocks access to the orthosteric ATP-binding pocket (Huo et al., 2018), these results suggest that the Ca^2+^ influx *via* transfected P2X4 receptors is inhibited by the presence of 5-HT_3_A receptors without interfering with the ATP binding site through direct steric interaction or *via* a downstream effect.

To test whether or not both P2X4 and 5-HT_3_A receptors interact with each other, tsA201 cells co-expressing P2X4 and 5-HT_3_A receptors were identified by confocal, TIRF and epifluorescence analysis using anti-HA and anti-MYC antibodies. As shown in Figures 2A–C, confocal imaging revealed that both receptors colocalize in the same cell at the plasma and intracellular membranes. Then, the colocalization of P2X4 and 5-HT_3_A receptor complexes at the plasma membrane was quantified using TIRF microscopy (Figures 2D–F) analysis, which showed a significant colocalization Pearson R index of 0.88 ± 0.09 (Figure 2G).

To evaluate a potential direct interaction, P2X4 or 5-HT_3_A receptors were purified with HA or Ni^2+^ bound agarose beads from tsA201 cells expressing only one type of receptor. The success and specificity of the purification process was confirmed by Western blot analysis against HA or MYC tag epitopes for P2X4 or 5-HT_3_A receptors, respectively (Figures 2J,L). Electrophoretic migration for each subunit (55 kDa for 5-HT_3_A and 70 kDa for P2X4) reached a similar molecular weight of that previously published (Barrera et al., 2005a) for 5-HT_3_A (55kDa) and slightly larger of that reported (Antonio et al., 2011) for P2X4 (65kDa). The difference observed in the electrophoretic migration of P2X4 subunit could correspond to a bigger glycosylation state, which is a posttranslational modification commonly observed for P2X receptors (Barrera et al., 2005c; Ormond et al., 2006; Antonio et al., 2011), since, according to its primary sequence, P2X4 subunit should have a molecular weight of 43.5 kDa. Once both P2X4 and 5-HT_3_A receptors were co-expressed in tsA201 cells, the purification process was based on pull-down experiments targeting the HA tag epitope in the P2X4 receptor. As a negative control, 5-HT_3_A receptors expressed alone were not detected in pull-down experiments using the HA-based purification protocol (data not shown). Western blot analysis demonstrated the presence of the P2X4 receptor in the eluted samples, but also a band corresponding to the 5-HT3A subunit was detected by anti-MYC antibody binding, demonstrating that both P2X4 and 5-HT_3_A receptors interact each other in cells co-expressing both receptors (Figure 2K). A similar finding was observed after pulling down the complex by targeting the His6 tag epitope in the 5-HT_3_A receptor, where a band corresponding to the P2X4 subunit was detected by anti-HA antibody binding (Figure 2M). To analyze the stoichiometry of the interaction, complexes formed by interacting P2X4/5-HT_3_A receptors were recorded by AFM imaging (Figures 3A–D). The molecular volumes of the purified samples showed 2 peaks at 489 ± 24 nm^3^ and 1100 ± 109 nm^3^, which are consistent with the detection of P2X4 alone (417 nm^3^; Antonio et al., 2011) and a P2X4/5-HT_3_A receptor complex with a stoichiometry 1:1; this is 417 nm^3^ and 757 nm^3^ (Barrera et al., 2005a; Figure 3B). After stimulation of the purified sample with 100 μM ATP in solution before adsorption onto mica for AFM imaging, the proportion of the complex marginally increased from 42% to 51% (area under the double Gaussian curve fit, cut off 800 nm^3^) with no change on the peak values (505 ± 15 nm^3^ and 1236 ± 62 nm^3^; *p* > 0.05; Figure 3D). All the peaks at histograms were derived from non-linear Gaussian distributions and were elected those that presented the best fits, R^2^ values corresponding to 0.9307 and 0.9126 for the P2X4/5-HT_3_A receptor complexes in the absence and presence of ATP, respectively.

To determine the protein concentration of the purified samples obtained from cells transfected with P2X4 or P2X4/5-HT_3_A receptor complexes, we used the number of protein particles adsorbed into mica, as an indicator of the concentration determined by the Avogadro Number relationship (equation 2). This analysis showed that P2X4 receptors from single transfected cells and P2X4/5-HT_3_A receptor complexes from cotransfected cells had a similar concentration of 3.3 ± 0.6 pM and 3.7 ± 1.3 pM (*p* > 0.05), respectively. However, AFM imaging of purified samples from cotransfected cells also presented an abundant peak at approximately the volume of individual P2X4 receptors, which suggests that pulling down the HA-tagged protein (P2X4 receptor) from the plasma membrane purified the complexes as well as P2X4 receptors alone and, therefore, increased the overall P2X4 expression at the plasma membrane. This correlates well with the TIRF microscopy analysis, where the P2X4 receptor expression in the cotransfected tsA201 cells was approximately two-fold higher than that observed on single transfected cells (Figure 2H). Interestingly, according to the analysis of epifluorescence imaging, the total cellular P2X4 receptor expression in tsA201 cells was similar in single and cotransfection experiments (Figure 2I), indicating a cellular redistribution of P2X4 receptors depending upon the presence of 5-HT_3_A receptors.

To calculate interaction energy between P2X4 and 5-HT_3_A receptors, macromolecular dockings for the interacting receptors were followed by 100 ns MD simulations, considering domains involved in the physical crosstalking between P2X and 5-HT_3_ receptors (Boué-Grabot et al., 2003; Emerit et al., 2016), and the larger interaction energy between membrane protein complexes observed through larger subunit interfaces (Casuso et al., 2012; Figures 3E,F). Total interaction energy (van der Waals and electrostatic energies) of P2X4 receptor at the *apo* state with 5-HT_3_A receptor was −301 ± 9 kcal/mol (mean ± SE; Figure 3E), which was not statistically different (*p* > 0.05) from that observed with P2X4 receptor at *holo* state (−278 ± 12 kcal/mol; Figure 3F). Also, the binding interaction energy of 3 ATP molecules to P2X4 receptor *holo* (−310 ± 12 kcal/mol) was significantly larger than that observed in the presence of interacting 5-HT_3_A receptor (−168 ± 3 kcal/mol, *p* < 0.05), suggesting that the inhibitory effect of 5-HT_3_A receptor could be allosteric, reducing the ATP affinity of the P2X4 receptor.

## Discussion

The P2X4 receptor activation by ATP binding triggered an initial transient increase in intracellular Ca^2+^ level that was followed by a slow component, where only the transient increase was strongly inhibited by the 5-HT_3_A receptor, which could correlate well with a reduced Ca^2+^ influx through P2X receptors. Our data brings a novel outcome for the P2X4/5-HT_3_A receptor complex interaction. A functional cross-inhibition has been demonstrated between P2X2 and 5-HT_3_A receptors (Boué-Grabot et al., 2003), but that study was based on simultaneous activation of the receptors by parallel stimulation with ATP and 5-HT. This current report demonstrates that the mere presence of the 5-HT_3_A interacting receptor in the complex, independent of its agonist, is sufficient to inhibit the P2X4 receptor. A similar inhibitory response has been observed for the mGlu5a receptor agonist-independent effect on the NMDA receptor (Perroy et al., 2008). Nevertheless, it remains to be determined whether the activation of the 5-HT_3_A receptor can further inhibit the P2X4 receptor-dependent ATP response or the P2X4 receptor *apo* could be able to inhibit the activated 5-HT_3_A receptor, which would enlighten the whole mechanistic scenario of these ionotropic receptors’ interaction.

Our data are consistent with the notion that P2X4/5-HT_3_A receptor interaction results in an allosteric inhibition of ATP-induced P2X4 activation. To further support this proposal, we performed additional experiments to reach the saturation of the concentration-response curve elicited by ATP in cells transfected with P2X4 receptor alone or co-transfected with P2X4 and 5-HT_3_A receptors. However, stimulation with 1 mM ATP induced an increase in intracellular Ca^2+^ concentration in non-transfected cells (mock cells), which precluded the direct comparison of the response observed at different ATP concentrations (data not shown), and then, the analysis of the Emax and EC50 observed in response to ATP, as required to confirm the presence of an allosteric interaction of 5-HT_3_A receptors with P2X4 receptors.

An alternative explanation to the inhibitory action of 5-HT_3_A receptors on the P2X4 receptor-triggered Ca^2+^ increase would be a reduction of the expression of P2X4 receptors at the plasma membrane in cotransfected cells. Conversely, we found a similar total expression of the P2X4 receptor between transfected tsA201 cells containing P2X4 and P2X4/5-HT3A subunits. Furthermore, the expression of P2X4 receptors specifically at the plasma membrane, revealed by TIRF microscopy analysis, indicated a two-fold increase of the purinergic receptor in cotransfected cells. Interestingly, a redistribution of P2X4 receptor from intracellular locations to the cell surface without changing total cellular expression has been demonstrated after activation of the C-C chemokine receptor type 2 in microglial cells, which is mediated by delivery of lysosomal P2X4 receptor to the plasma membrane (Toyomitsu et al., 2012). Besides, the P2X4 receptor has been shown to colocalize with the 5-HT_3_A receptor at the cell surface of hippocampal neurons when its internalization motif is replaced by a FLAG epitope (Emerit et al., 2016). Our results have shown that this mutation is not needed for a significant plasma membrane expression of both receptors; however, it has yet to be determined whether the physical interaction between P2X4 and 5-HT_3_A receptors is enough to trigger the protein trafficking or requires intracellular signaling pathway. Remarkably, the expression pattern of purified P2X4/5-HT_3_A receptor complexes vs. the P2X4 receptor alone, revealed by AFM imaging, resembles relatively well to that observed on the plasma membrane in living cells. Almost half of the particles of the purified samples from tsA201 cells coexpressing P2X4 and 5-HT_3_A receptors correspond to the assembled complex and present an identical concentration to that from cells expressing only P2X4 receptors. If we consider the other half of particles that match the molecular volume of free P2X4 receptors, then the total purinergic receptor should increase almost twice, reinforcing our notion that the purification process is highly enriched in the plasma membrane fraction.

Our AFM results demonstrate for the first time that the P2X4/5-HT_3_A receptor complex is formed with a stoichiometry 1:1, which is maintained by the presence of the P2X4 receptor agonist. To propose a molecular way for the allosteric inhibition of 5-HT_3_A receptor on P2X4 receptor function, we embarked on testing a docking between both receptors, considering the postulated domains for physical crosstalking (Boué-Grabot et al., 2003; Emerit et al., 2016), and interfaces with larger surfaces that maintain a trimeric/pentameric symmetry, which has been already postulated as variables energetically favorable for interacting macromolecules (Casuso et al., 2012). After 100 ns MD simulations, no differences in interaction energy between the P2X4 receptor *apo* or P2X4 receptor *holo* complexed with 5-HT_3_A receptors were found. Also, the presence of the interacting 5-HT_3_A receptor decreased the ATP binding interaction energy of ATP molecules to the P2X4 receptor, which is consistent with a negative allosteric effect of 5-HT_3_A receptors that preserves the 1:1 stoichiometric interaction between both receptors. Nevertheless, further bioinformatic analysis should be performed to corroborate these findings, in particular, to explore extensively other receptor-receptor surface dockings *via*, for example, coarse-grained approaches, which would result in the same or additional conformations of the receptor complex.

Crosstalking mechanisms lead to more complex neuronal signaling by providing additional regulatory roles of agonists and receptors. Taken together, we have shown here that the P2X4 receptor is inhibited by a physical interaction with the 5-HT_3_A receptor in a 1:1 stoichiometry and ATP maintains this complex. As 5-HT_3_A receptor can interact with other P2X receptors subtypes such as P2X2 and P2X3 (Emerit et al., 2016), it remains to be solved if the same stoichiometry and steric mechanism participate in the functional regulation of these receptors. Furthermore, as P2X4 receptors can be modulated by membrane phosphoinositides (Bernier et al., 2008), it could be further explored whether or not the 5-HT_3_A receptor and phospholipids can coexist to control the P2X4 receptor function *via* modifications of the stoichiometric binding.

## Data Availability Statement

All datasets generated for this study are included in the article.

## Author Contributions

PS performed AFM experiments and data analysis, immunofluorescence and western blot experiments. PG performed Ca^2+^ measurements and helped writing the article. CF performed homology modeling, molecular docking, and data analysis. BL performed western blot and TIRF experiments, followed by a quantitative analysis of immunofluorescence images. PN performed western blot and TIRF experiments. XF supervised Ca^2+^ measurements and helped writing the article. NB designed and supervised the investigation, analyzed the data and wrote the article.

## Conflict of Interest

The authors declare that the research was conducted in the absence of any commercial or financial relationships that could be construed as a potential conflict of interest.
